# Promotion of a Mediterranean Diet Alters Constipation Symptoms and Fecal Calprotectin in People with Parkinson’s Disease: A Randomized Controlled Trial

**DOI:** 10.3390/nu16172946

**Published:** 2024-09-02

**Authors:** Carley Rusch, Matthew Beke, Carmelo Nieves, Volker Mai, Tamara Stiep, Tracy Tholanikunnel, Adolfo Ramirez-Zamora, Christopher W. Hess, Bobbi Langkamp-Henken

**Affiliations:** 1 Food Science and Human Nutrition Department, Center for Nutritional Sciences, University of Florida, Gainesville, FL 32611-0370, USA; carley.rusch@abbott.com (C.R.); bekematthew@ufl.edu (M.B.);; 2 Department of Neurology, Norman Fixel Institute for Neurological Diseases, University of Florida, Gainesville, FL 32610-0236, USA; tamara.stiep@ucsf.edu (T.S.); tracy.tholanikunnel@neurology.ufl.edu (T.T.); adolfo.ramirez-zamora@neurology.ufl.edu (A.R.-Z.); christopher.hess@neurology.ufl.edu (C.W.H.); 3 Department of Epidemiology, Emerging Pathogens Institute, University of Florida, Gainesville, FL 32610-0009, USA; vmai@epi.ufl.edu

**Keywords:** Parkinson’s disease, Mediterranean diet, constipation, microbiota, inflammation

## Abstract

Parkinson’s disease is associated with gastrointestinal (GI) dysfunction, including constipation symptoms and abnormal intestinal permeability and inflammation. A Mediterranean diet (MediDiet) may aid in disease management**.** This parallel, randomized, controlled trial in people with Parkinson’s (PwP) and constipation symptoms compared a MediDiet against standard of care on change in constipation symptoms, dietary intake, and fecal zonulin and calprotectin concentrations as markers of intestinal permeability and inflammation, respectively. Participants were randomized to either standard of care for constipation (control; *n* = 17, 65.1 ± 2.2 years) or a MediDiet plus standard of care (*n* = 19, 68.8 ± 1.4 years) for 8 weeks. Constipation scores decreased with both interventions (*p* < 0.01), but changes from baseline were not different between groups (MediDiet, −0.5 [−1.0, 0]; control, −0.8 [−1.0, 0.2]; median [25th, 75th]; *p* = 0.60). The MediDiet group had a higher intake of dietary fiber at week 4 than the control group (13.1 ± 0.7 g/1000 kcal vs. 9.8 ± 0.7 g/1000 kcal; *p* < 0.001). No differences in fecal zonulin were observed between groups (*p* = 0.33); however, fecal calprotectin tended to be lower in the MediDiet group at week 8 (45.8 ± 15.1 µg/g vs. 93.9 ± 26.8 µg/g; *p* = 0.05). The MediDiet and standard interventions reduced constipation symptoms; however, the MediDiet provided additional benefit of increased dietary fiber intake and less intestinal inflammation.

## 1. Introduction

Parkinson’s disease, the second most common neurodegenerative disease [1,2], is associated with pathological aggregation of alpha-synuclein, which may lead to dopaminergic loss in the brain. More recent evidence suggests there may be a gut-to-brain spread of alpha-synuclein via the vagus nerve in animal models, suggesting Parkinson’s disease may have origins in the gastrointestinal (GI) tract [3,4,5]. Despite this, little is known about the impact of different diets on symptoms and disease progression. Most people with Parkinson’s (PwP) experience GI symptoms affecting the entirety of the GI tract, such as abnormal salivation, dysphagia, weight loss, gastroparesis, constipation, and defecatory symptoms. Symptoms might precede the onset of motor concerns and diagnosis of the disease [3]. Constipation, one of the most prominent GI symptoms [6], is considered a prodromal marker [7] and is associated with decreased quality of life (QOL) in PwP [8].

The term “constipation” is a generalization that typically involves either acute symptoms of difficult, infrequent, or incomplete defecation that last less than a week or chronic (occurrence ≥ 3 months) symptoms [9]. Stool frequency (<3 bowel movements per week) and form are used in practice either alone or in combination as part of a comprehensive exam (i.e., Rome IV Criteria) to categorize constipation symptoms [10]. Additionally, the Gastrointestinal Symptom Rating Scale (GSRS) is a validated, clinical rating scale that captures bothersome constipation symptoms (hard stools, constipation, incomplete emptying) as well as other GI syndromes such as diarrhea, abdominal pain, indigestion, and reflux [11]. Once identified, treatment of constipation symptoms includes lifestyle modifications such as increasing exercise, fluids, and dietary fiber intake [12].

Constipation is associated with microbial alterations and increased intestinal inflammation and permeability, which may be potentiated in times of chronic illness due to interactions with innate and adaptive immune systems [13,14]. Interestingly, Parkinson’s disease has been associated with markers of intestinal permeability (e.g., orally administered sugar probes and fecal zonulin), inflammation (e.g., fecal calprotectin), and a dysregulated microbiota that may impact disease progression [3,15,16,17]. Further, alpha-synuclein has been documented along the GI tract, including the colon, implicating its role in the pathogenesis of GI dysfunction in Parkinson’s disease [18]. This relationship has further sparked an interest in elucidating the connection of the gut–brain axis in PwP for early detection and neuroprotective therapies [19].

The Mediterranean diet (MediDiet) is a potential neuroprotective dietary pattern [20,21] that encourages consumption of colorful fruits, vegetables, legumes, unrefined cereals, and fermented milk products [22]. These foods provide a source of antioxidants, dietary fibers, and polyphenolic compounds for intestinal bacteria, which may be selectively fermented to produce short-chain fatty acids (SCFA) such as acetate, butyrate, and propionate. These SCFA are important for intestinal barrier function and inflammation [23,24]. Higher adherence to the MediDiet has been associated with increased microbial richness and a higher abundance of beneficial bacteria and SCFA [25,26,27,28,29,30]. Studies demonstrate alterations in intestinal bacteria and bacterial products in PwP, such as decreased *Faecalibacterium* (butyrate producer), *Prevotellaceae*, and SCFA compared to healthy controls [19,31,32,33,34,35]. To date, there have been limited dietary interventions studied to specifically improve motor and non-motor symptoms of Parkinson’s disease [30]. We completed a single-arm pilot study (*n* = 8), which demonstrated feasibility of a MediDiet, improvement in constipation symptoms, and beneficial changes to microbes in PwP [36]. Because the study was limited by the lack of a control group and small sample size, further study was warranted. Therefore, the overall purpose of this randomized, controlled trial was to determine the effect of a MediDiet intervention in PwP on GI function (e.g., constipation symptoms), markers of intestinal inflammation and permeability, and fecal microbial communities.

## 2. Materials and Methods

### 2.1. Study Design and Participants

The MEDI-PD study was a randomized controlled parallel study in PwP who experience constipation symptoms (Clinical Trial #NCT04683900). This study was conducted at a site in the Southeastern United States from February 2021 through June 2022. Protocol details of the MEDI-PD study have been previously described in detail [37]. In brief, PwP who experienced constipation symptoms were recruited. Additionally, to be eligible, participants had to be drug-naïve or on a stable regimen of Parkinson’s medications with a Hoehn and Yahr (H&Y) stage of ≤2.5 in the clinical ‘on’ state and consume <20 g of fiber per day. Participants were not eligible if they had atypical or secondary parkinsonism or a physician-diagnosed GI disease, were underweight (body mass index < 18.5 kg/m^2^), or had a history of deep brain stimulation surgery. Participants provided informed consent at a virtual visit and enrolled in the 10-week study. The study consisted of a 2-week baseline period and an 8-week intervention to compare the standard of care for constipation (control group) to the standard of care plus a Mediterranean diet (MediDiet group). During the 2-week baseline period, participants completed questionnaires and were instructed to maintain their usual diet. Participants attended the first study visit during the baseline period (week 0) and completed nutritional and neurological evaluations. Participants were stratified by sex and H&Y stage (0 to 1.5 or 2 to 2.5) and randomized via sealed envelopes in a 1:1 allocation to the control or MediDiet intervention [37]. Participants in the control group were provided a handout on recommendations for managing constipation symptoms, which included recommendations for increasing physical activity and fluid and dietary fiber intake and laxative usage (standard of care). Those randomized to the intervention group were provided the same constipation handout and counseled on the MediDiet. Guidelines for the diet followed the PREDIMED trial protocol [38,39]. Following the 2-week pre-baseline period, all participants were asked to begin incorporating the dietary recommendations into their daily routine (day 1). Participants returned to the study site after 4 and 8 weeks and repeated study measurements. Due to the COVID-19 pandemic, the study format was adjusted to run the study online for interested participants who qualified. Informed consent and all questionnaires were completed online as outlined in the original protocol [37]. Eligibility was verified after informed consent using medical records, including the H&Y stage. Study materials, including stool collection kits, were mailed to participants. Participants in the virtual group participated in the study visits using video conferencing. This study was conducted in accordance with the guidelines laid down in the Declaration of Helsinki, and all study-related procedures were approved and monitored by the University of Florida Institutional Review Board-01.

### 2.2. Questionnaires and Nutritional and Neurological Evaluations

As previously described, participants completed daily and weekly questionnaires during the 2-week pre-baseline period and throughout the entire 8-week intervention [37]. Daily questionnaires inquired about GI function, including stool frequency and form (Bristol Stool Form Scale, BSFS), laxative usage, perceived stress (0 = no stress to 10 = extremely stressed), and adverse medical events [40]. Weekly questionnaires assessed GI symptoms using the GSRS, the Digestion-associated Quality of Life Questionnaire (DQLQ), and adherence to the Mediterranean diet [11,41]. The GSRS assesses the severity of 15 GI symptoms grouped into 5 syndromes (i.e., constipation, diarrhea, indigestion, abdominal pain, and reflux syndromes). The DQLQ consists of 9 statements scored 0 = never to 1.0 = always regarding how often digestive events affected QOL. The total score ranges from 0 = better to 9 = worse digestion-associated QOL. The 14-point Mediterranean Diet Adherence Screener (MEDAS) was used to assess adherence to the Mediterranean diet [42,43]. The Automated Self-Administered 24-h Dietary Recall (ASA-24) was administered on 4 different days of each study visit week to assess energy and nutrient intake, including dietary fiber intake [44]. The International Physical Activity Questionnaire, which was administered prior to each study visit, was used to assess metabolic equivalent task (MET) minutes of activity [45].

Because the adoption of a MediDiet may promote a calorie deficit that is associated with weight loss in previous studies [46], nutritional status was monitored throughout the study as weight loss is associated with faster cognitive decline and increased Movement Disorder Society Unified Parkinson’s Disease Rating Scale (MDS-UPDRS) scores in PwP [47]. For participants who attended study visits in person, body composition and handgrip strength were assessed using bioimpedance spectroscopy (XiTRON Hydra 4200) and a dynamometer (Jamar Plus Digital Hand Dynamometer), respectively, and a study dietitian assessed overall nutritional status using the Patient-Generated Subjective Global Assessment (PG-SGA) [48]. For online participants, body composition, PG-SGA, and handgrip strength assessments were not completed, and height and weight were self-reported.

Study neurologists evaluated baseline motor and non-motor symptoms (MDS-UPDRS) and cognitive function (Montreal Cognitive Assessment [MoCA]) [49,50] in participants who attended in-person study visits. As exploratory variables, symptoms of depression, anxiety, and quality of life were evaluated using the Hamilton Depression (HAM-D) and Anxiety Scales (HAM-A) and the Parkinson’s Disease Questionnaire-39 (PDQ-39) [51,52,53].

### 2.3. Intestinal Permeability and Inflammatory Markers

Fecal samples were collected at baseline, week 4, and week 8 and stored according to study protocol [37]. Fecal zonulin and calprotectin were measured as an indicator of intestinal permeability [54] and inflammation [55]. Both biomarkers were quantitated using an ELISA (Calprotectin ELISA Assay Kit, Eagle Biosciences; IDK Zonulin ELISA Kit, Immundiagnostik) according to manufacturer protocols.

### 2.4. Fecal Microbiota Analyses Based on 16S rDNA Sequencing

For fecal microbial analyses, samples were collected at baseline, week 4, and week 8 and stored at −20 °C before DNA extraction using a standard protocol (QIAamp DNA Mini Kits; Qiagen, Germantown, MD, USA) with an additional bead-beating step with 0.1 mm diameter zirconia beads (BioSpec, Bartlesville, OK, USA) to mechanically disrupt bacterial cell walls. DNA concentration in the final extraction volume of 100 µL was measured using Nanodrop Nucleic Acid Quantification (Thermo Fisher, Waltham, MA, USA). DNA was amplified by PCR (High-Fidelity Hot Start Taq 2X Master Mix 3, New England BioLabs, Ipswich, MA, USA) with primers MiSeq 27F and MiSeq-338R (Illumina, Inc., San Diego, CA, USA) containing unique barcodes. Amplification was verified by gel electrophoresis, and amplicons were purified using Mag-Bind TotalPure (Omega Bio-Tek, Norcross, GA, USA). Amplicon DNA concentration was measured through the Quant-iTTM dsDNA Assay kit (Invitrogen, Carlsbad, CA, USA). DNA was cleaned and pooled in equimolar amounts for sequencing on a 2x250xv2 MiSeq Nano flow cell with an 8pM loading concentration and a 25% PhiX spike (MiSeq; Illumina, Inc. through the University of Florida ICBR).

### 2.5. Statistical Methods

#### 2.5.1. Sample Size Calculation and Primary Outcome

The sample size for the primary outcome was calculated based on changes in constipation symptoms from baseline to final in the pilot study, which were assessed using the mean GSRS constipation syndrome score from 3 symptoms (i.e., constipation, hard stools, and the feeling of incomplete evacuation) scored 1 = no discomfort to 7 = very severe discomfort [36]. A sample size of *n* = 17 per intervention group was required to observe a mean difference of 0.7 ± 0.7 (±SD; *p* < 0.05; 80% power; 0.99 effect size) in the change in constipation syndrome score between interventions [37]. In the current study, the change in GSRS constipation syndrome scores (mean of weeks 7 and 8 minus the mean of weeks −1 and 0) were not normally distributed, thus the comparison between intervention groups was analyzed using a nonparametric Mann–Whitney U test (SAS Institute Inc., version 9.4, Cary, NC, USA).

#### 2.5.2. Questionnaires and Nutritional and Neurological Evaluations

Questionnaires were analyzed on an intent-to-treat (ITT) basis. Data from weeks 0, 4, and 8 were analyzed using a generalized linear mixed model (GLMM) with intervention group, week, and the interaction of group and week as fixed effects with the random effect of subject to account for the repeated measures. Covariate analyses included age, sex, H&Y stage, fiber intake, MET minutes, laxative usage, bowel movement frequency, and stress in initial models. Nonsignificant covariates were removed hierarchically beginning with interactions with the largest *p*-values. Post hoc tests with the use of the Tukey–Kramer method for multiple comparisons were conducted on questionnaire outcomes that had significant effects. In the case of missing data (i.e., non-compliance and/or withdrawals), the last available data were carried forward to week 8 for ITT analyses. Questionnaire data from 2 participants in the control group were carried from week 4 to 8 due to missing data for ITT analyses. Per protocol (PP) analyses were completed as indicated for participants who completed study procedures and scored >10 for the MediDiet group (*n* = 12) and <10 for the control group (*n* = 11) based on the 14-point MEDAS at 4 and 8 weeks. Continuous data are reported as means ± SEM. A type I error rate cutoff of 0.05 was used to denote statistical significance. Categorical variables (demographic and PG-SGA stages) are shown as frequencies or proportions. Normality was confirmed using histogram frequencies and QQ plots of residuals. If normality could not be assumed, then data were log transformed or a non-parametric test was used. Data were analyzed using a statistical software program (SAS Institute Inc., version 9.4, Cary, NC, USA).

Data from daily questionnaires were converted to weekly sums or averages. Weekly stool frequency and laxative usage were calculated by adding the number of bowel movements or laxative days each week. Stool form and perceived stress were assessed at each study week using the average weekly BSFS and perceived stress score, respectively. Weekly GSRS syndrome scores, DQLQ scores, stool frequency, weekly percentage of hard stools (calculated using the BSFS), laxative usage, physical activity scores, and perceived stress were log-transformed for analysis to meet the assumptions of normality and homogeneity of variance. The 4 dietary recalls (2 weekdays and 2 weekend days) from weeks 0, 4, and 8 were averaged and then analyzed on an ITT basis using GLMM with group, week, total energy intake, and group and week interactions in the final GLMM model (unless otherwise noted) and adjusted using the post hoc Tukey–Kramer method for multiple comparisons. Dietary fiber was analyzed as total intake (grams) and density (grams per 1000 kcal) using GLMM (as described above). Exploratory questionnaires, including Parkinson’s-associated QOL, anxiety, and depression questionnaires, as well as body composition analyses, are described in Supplementary Methods.

#### 2.5.3. Intestinal Permeability and Inflammatory Marker Analyses

A GLMM was used to analyze concentrations of fecal zonulin and calprotectin. Baseline (week 0) values were taken out of the model and instead used as a covariate, with only intervention weeks 4 and 8 in the final model. Baseline values were compared using a Mann–Whitney U test to see if any differences existed between groups. Both markers were log-transformed for analysis to meet the assumptions of normality and homogeneity of variance. Additional covariates were included in the full models, with nonsignificant covariates removed hierarchically, beginning with interactions with the largest *p-*values. For ITT analyses, missing data for week 8 were carried forward from week 4 from 4 participants in the control group for fecal zonulin as well as 2 participants in the control group for fecal calprotectin. PP analyses were conducted on eligible participants as described above (MediDiet group, *n* = 12; control group, *n* = 11). Data are reported as mean ± SEM. A type I error rate cutoff of 0.05 is used to denote statistical significance.

#### 2.5.4. Fecal Microbiota Analysis Based on 16S rDNA Sequencing

Sequencing reads were analyzed first using the Quantitative Insights into Microbial Ecology 2 tool (version 2020.11) pipeline [56]. Quality and adapter trimming were performed using CutAdapt [57,58]. Further de-noising and amplicon sequence variant (ASV) calling were performed using Deblur [59]. ASVs were aligned with MAFFT [60], and a phylogenetic tree for diversity analyses was generated with the FastTree pipeline [61]. ASVs were clustered into de novo operational taxonomic units (OTUs) at the 98% similarity level using VSEARCH [62]. Taxonomy was assigned to OTUs using a classifier built with Scikitlearn [63] and verified against the Greengenes 16S rRNA gene database (Version 13.8) [64]. Rarefaction curves were used to assess species richness and comparability of the samples. Microbiomes between interventions were compared by alpha diversity index (Shannon index) and beta diversity (Unifrac and Weighted Unifrac). Differential abundance analyses were performed using Phyloseq [65] and edgeR [66,67] packages, using R version 4.0.5 and the Rstudio interface.

## 3. Results

### 3.1. Participant Characteristics and Compliance

**Figure 1:**
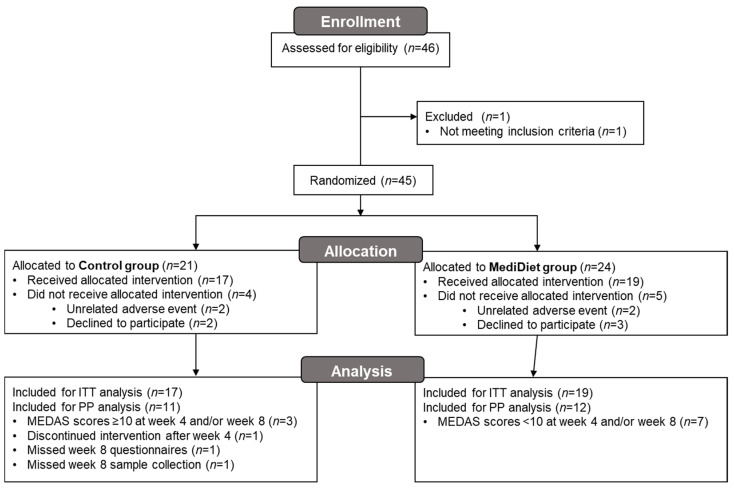
Participant flow diagram for the main study. Due to the coronavirus pandemic, 11 participants (*n* = 3 control group and *n* = 8 MediDiet group) completed study procedures virtually. Abbreviations: ITT, intent-to-treat; MEDAS, Mediterranean diet adherence screener; MediDiet, Mediterranean diet; PP, per protocol.

After randomization, four participants withdrew from the control group and five participants withdrew from the MediDiet group before receiving the allocated intervention (Figure 1). Due to the coronavirus pandemic, 11 participants (*n* = 3 control group and *n* = 8 MediDiet group) completed study procedures virtually. All participants provided week 0 and 4 stool samples. Two participants in the control group did not provide the week 8 sample. Of these participants, one withdrew after week 4 due to a hospitalization for confusion and pneumonia but was included in the ITT analyses. The second participant completed the study but did not provide a stool. For PP analyses, six participants were excluded in the control group due to missing week 8 questionnaires and stool samples, high adherence to a MediDiet, or an unrelated adverse event.

**Figure 2:**
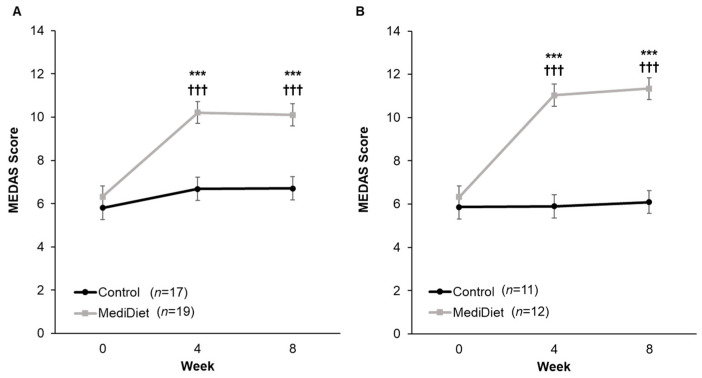
Mediterranean diet adherence by study week from the 14-Item Mediterranean Diet Adherence Screener (scores from 10 to 14 denote good adherence). The interaction of group and week was significant for both intent-to-treat ((**A**), *p* = 0.002) and per protocol ((**B**), *p* < 0.0001). A general linear mixed model was used to analyze scores. Covariates were tested in the full model, and nonsignificant covariates were removed hierarchically, beginning with interactions with the largest *p*-values. *p*-values were adjusted using the post hoc Tukey–Kramer method for multiple comparisons. *** *p*-value < 0.001 MediDiet vs. control groups at specified week. ^†††^ *p*-value < 0.001 compared to week 0 in the MediDiet group. Values are least squares means ± SEM. Abbreviations: MEDAS, Mediterranean diet adherence screener; MediDiet, Mediterranean diet.

Adherence to a MediDiet at week 0 was low (MEDAS score < 10 indicating poor adherence) and not different between groups (*p* = 0.984, Figure 2). For the ITT analysis, there was a significant interaction of group and week (*p* = 0.0002) whereby MEDAS scores were higher in the MediDiet group at weeks 4 and 8 than the control group (*p* < 0.001 and *p* < 0.001, respectively; Figure 2A). MEDAS scores were not different at weeks 4 and 8 (*p* = 0.532 and *p* = 0.714, respectively) compared to week 0 in the control group. In the MediDiet group, seven participants were excluded from PP analyses due to low adherence to the MediDiet intervention. MEDAS scores between PP intervention groups at weeks 4 and 8 were significantly different (*p* < 0.001; Figure 2B).

Baseline demographics were not different between the intervention groups, except for the mean baseline MoCA score, which was below the cutoff score for normal cognitive function (i.e., ≥26) and lower in the MediDiet group versus the control group (*p* = 0.023; Table 1) [50]. Most participants were white males and non-Hispanic or Latino and had an H&Y stage 2.0–2.5 with an average disease duration of approximately 5 years since clinical diagnosis. No adverse events reported were attributed to the study interventions or procedures.

### 3.2. Primary Outcome

**Figure 3:**
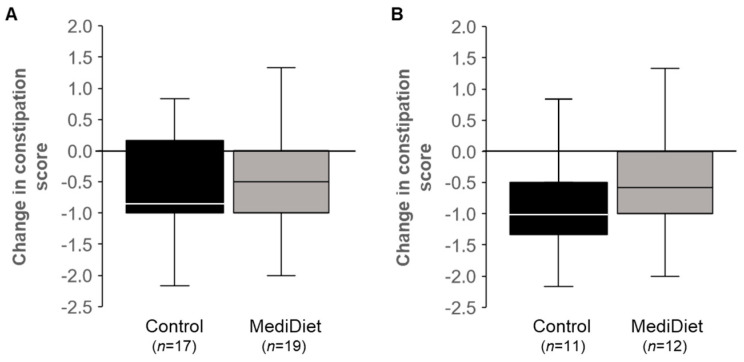
Constipation syndrome scores from the Gastrointestinal Symptom Rating Scale decreased in both groups (*p* < 0.05) over the 8-week intervention. The constipation symptoms (constipation, hard stools, and the feeling of incomplete evacuation) are scored as 1 = no discomfort to 7 = very severe discomfort, with constipation syndrome representing the mean symptoms score. Data did not meet the assumptions of normality, and Mann–Whitney U tests were used. No differences between groups were observed for intent-to-treat ((**A**), *p* = 0.600) or per protocol ((**B**), *p* = 0.147) analyses. Median scores are represented by the line within each boxplot. Abbreviations: MediDiet, Mediterranean diet.

Mean constipation symptom scores decreased (i.e., negative change; *p* < 0.01) with both; however, the change was not different between groups for ITT (*p* = 0.600; Figure 3A) or PP (*p* = 0.147; Figure 3B).

### 3.3. Questionnaire Data

#### 3.3.1. Gastrointestinal Function

**Figure 4:**
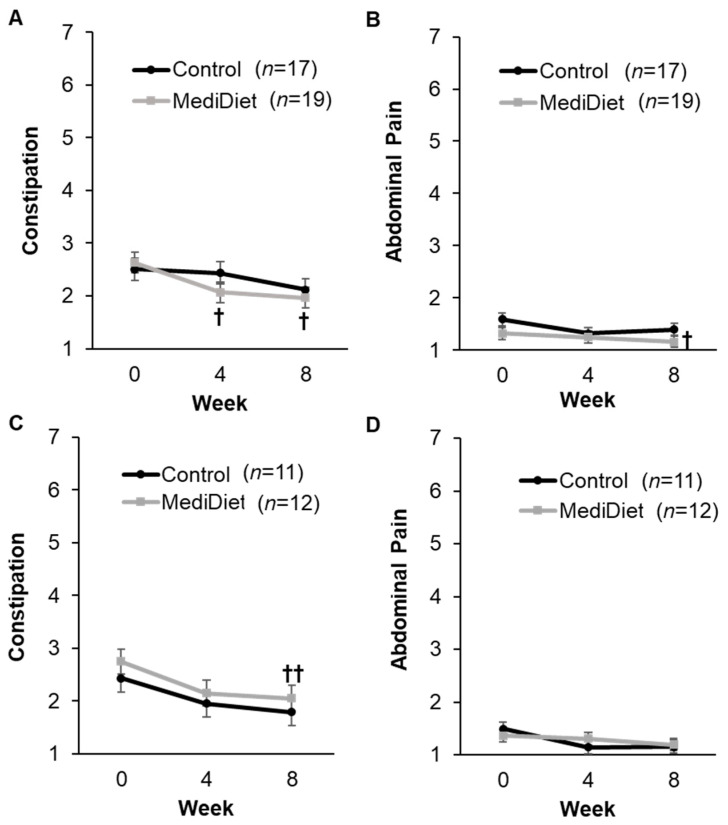
Syndrome scores from the Gastrointestinal Symptom Rating Scale by study week in the intent-to-treat (**A**,**B**) and per protocol analyses (**C**,**D**). The constipation syndrome score (**A**,**C**) includes constipation, hard stools, and the feeling of incomplete evacuation. The abdominal pain syndrome score (**B**,**D**) includes abdominal pain, hunger pains, and nausea. Symptom scores range from 1 = no discomfort to 7 = very severe discomfort, with syndrome scores representing the mean symptom score. A generalized linear mixed model was used for analyses. Covariates were tested in the full model, and nonsignificant covariates were removed hierarchically, beginning with interactions with the largest *p*-values. Study week was significant for constipation ((**A**), *p* = 0.007; (**C**), *p* = 0.002) and abdominal pain ((**B**), *p* = 0.003; (**D**), *p* = 0.036) syndrome scores. Abdominal pain syndrome scores differed with the level of physical activity for intent-to-treat analyses ((**B**), *p* = 0.018) and were included as a covariate. *p-*values were adjusted using the post hoc Tukey–Kramer method for multiple comparisons. ^†^ *p*-value < 0.05 compared to week 0 in the MediDiet group. ^††^ *p*-value < 0.01 compared to week 0 in the MediDiet group. Scores were log-transformed for analyses to meet the assumptions of normality. Values are presented as untransformed least squares means ± SEMs. Abbreviations: MediDiet, Mediterranean diet.

GSRS constipation syndrome scores were significantly correlated with laxative usage (*r* = 0.348, *p* < 0.001) and stress (*r* = 0.283, *p* = 0.003). Therefore, the latter two covariates were not included in models assessing group effects on weekly GSRS constipation syndrome scores. There was a significant effect of study week (ITT, *p* = 0.007; PP, *p* = 0.002) but not for group (ITT, *p* = 0.581; PP, *p* = 0.373) or group and week interactions (ITT, *p* = 0.202; PP, *p* = 0.939; Figure 4A,C) on weekly GSRS constipation syndrome scores. Scores were highest at week 0 and their lowest at week 8, as expected, indicating constipation symptoms improved over the course of the study. Only the MediDiet group reported significantly decreased constipation scores at weeks 4 and 8 when compared to week 0 for ITT analyses (*p* = 0.041 and *p* = 0.039, respectively; Figure 4A). For PP analysis, only week 8 remained significantly decreased when compared to week 0 in the MediDiet group (*p* = 0.009; Figure 4C). The effect of study week was also significant for GSRS abdominal pain (ITT, *p* = 0.003; PP, *p* = 0.036; Figure 4B,D) and ITT reflux (*p* = 0.049) and PP diarrhea scores (*p* = 0.040; Supplementary Table S1). There was no effect of group or the interaction between the group and study week for the other GSRS syndrome scores (Supplementary Table S1).

Laxative usage was lower in the MediDiet group (*p* = 0.044) across all weeks and tended to decrease in both groups by study week (*p* = 0.055) after adjusting for age (*p* = 0.020) for the ITT analysis (Table 2). However, there was no group by week interaction. Results were similar for PP analysis after adjusting for sex (*p* = 0.045) and H&Y stage (*p* = 0.017). Stool frequency, percentage of hard stools, and digestion-associated QOL were not different within or between groups (Table 2).

#### 3.3.2. Dietary Intake

**Figure 5:**
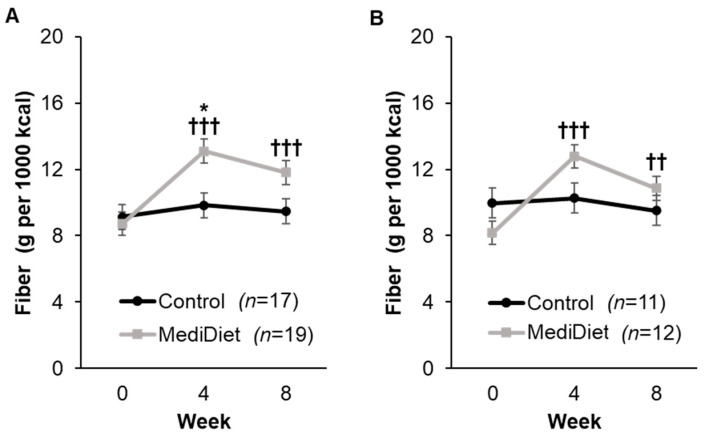
Dietary fiber intake density by study week as measured by the Automated Self-Administered 24-h recall. A general linear mixed model was used to analyze scores for ITT (**A**) and PP (**B**) analyses. Covariates were tested in the full model, and nonsignificant covariates were removed hierarchically, beginning with interactions with the largest *p*-values. The final model for the included group (*p* = 0.056), week (*p* < 0.0001), and interaction of group and week (*p* = 0.0007) for ITT analyses (**A**). For PP analyses (**B**), the final model included group (*p* = 0.553), week (*p* = 0.0002), and interaction of group and week (*p* = 0.0006) and was adjusted using post hoc Tukey–Kramer method for multiple comparisons. * *p*-value < 0.05 MediDiet vs. control groups at specified week. ^††^ *p*-value < 0.01 compared to week 0 in the MediDiet group. ^†††^ *p*-value < 0.001 compared to week 0 in the MediDiet group. Values are least squares means ± SEM of 4, 24-h recalls (2 weekday, 2 weekend) for each time point. Abbreviations: ITT, intent-to-treat; MediDiet, Mediterranean diet; PP, per protocol.

At baseline, there were no differences in dietary intake between groups for ITT analyses, including total energy intake (*p* = 1.000, Supplementary Table S2), after adjusting for sex (*p* = 0.0002). After the intervention period, there were no within- or between-group changes in intake of total calories, protein, carbohydrates, sugars, total fat, and water for ITT analyses. For dietary fiber intake density (g per 1000 kcal), there was a significant interaction of group and week (*p* = 0.0007, Figure 5A), whereby dietary fiber intake density was higher in the MediDiet group at week 4 (*p* = 0.027) but not different at week 8 (*p* = 0.221) when compared to the control group for ITT analyses. When compared to week 0 in the MediDiet group, dietary fiber intake density was increased at week 4 and week 8 (*p* < 0.0001 and *p* = 0.0002, respectively; Figure 5A). For PP analyses, the interaction for group and week remained significant (*p* = 0.0006, Figure 5B), but there was no difference between groups at week 4 and week 8 (*p* = 0.379 and *p* = 0.901, respectively). Within-group changes in the MediDiet group for dietary fiber intake density were similar to ITT for PP analyses (Figure 5B).

There was a significant interaction of group and week for total saturated fat (*p* = 0.004), total monounsaturated fat (*p* = 0.007), and oleic acid (*p* = 0.004; Supplementary Table S2) in the ITT analyses. Saturated fat intake decreased at weeks 4 (*p* < 0.0001) and 8 (*p* = 0.004) in the MediDiet group when compared to week 0 but did not differ from the control group (*p* = 0.759 and *p* = 0.585, respectively). Intake of monounsaturated fat was higher in the MediDiet group at week 4 (*p* = 0.0004) and tended to be higher at week 8 (*p* = 0.088) when compared to the control group. Within-group differences for the MediDiet group were only observed at week 4 (*p* = 0.0004) but not at week 8 (*p* = 0.226). Similarly, oleic acid was highest at week 4 (*p* = 0.0003) and trending higher at week 8 (*p* = 0.087) in the MediDiet group compared to controls. While there was a significant group by week interaction for total polyunsaturated fat (*p* = 0.019) and linoleic acid (*p* = 0.012), there were no significant differences between groups for total polyunsaturated fat and linoleic acid. Other differences in fatty acids, such as eicosapentaenoic and docosahexaenoic acid, were not observed between MediDiet and control groups (Supplementary Table S2).

#### 3.3.3. Nutritional Status and Body Composition

At baseline, body weight (ITT and PP, *p* = 1.00) and BMI (ITT; *p* = 0.998; PP, *p* = 0.996) were not different between groups. Models for body weight were not significantly different across all outcomes but differed by sex (ITT, *p* < 0.001; PP, *p* = 0.006) in which females had lower body weight than males (Supplementary Figure S1A,C). On average, participants in both groups were overweight at baseline (BMI > 25.0). BMI was significant for study week (ITT, *p* < 0.001; PP, *p* = 0.001) and interaction of group and week (ITT, *p* = 0.016; PP, *p* = 0.044; Supplementary Figure S1B,D).

For participants who completed in-person study visits (*n* = 25), all were classified as SGA stage A (well nourished) at baseline and throughout the study intervention period. Handgrip strength tended to differ with age (*p* = 0.051) and differed with sex (and *p* < 0.001; Supplementary Table S3), in which females and increasing age had lower handgrip strength as expected. Handgrip strength and body composition (fat-free and fat-mass percentages and total body water) were not different between treatment groups (Supplementary Table S3).

#### 3.3.4. Physical Activity and Perceived Stress

At baseline, self-reported physical activity, as measured by total MET minutes per week, was not different between treatment groups (MediDiet, 2785 ± 526; control, 2780 ± 518; *p* = 0.999) for ITT analyses. Further, no differences in physical activity were observed at week 4 (MediDiet, 4082 ± 1369; control, 3733 ± 766; *p* = 1.000) or week 8 (MediDiet, 3746 ± 1261; control, 3654 ± 1019; *p* = 1.000) between groups. For PP analyses, results were similar, and no differences between groups were observed.

Perceived level of stress was not different between groups at baseline (ITT; MediDiet, 2.3 ± 0.4; control, 3.2 ± 0.4; *p* = 0.820). There were no significant differences at week 4 (ITT; MediDiet, 2.2 ± 0.4; control, 3.2 ± 0.4; *p* = 0.530) or week 8 (ITT; MediDiet, 2.4 ± 0.5; control, 3.1 ± 0.3; *p* = 0.692) between groups. No within-group differences were observed across study weeks in the final models for ITT or PP analyses.

#### 3.3.5. Parkinson’s-Related Quality of Life, Anxiety, and Depression

For the bodily discomfort dimension, the control group reported higher scores indicating worse QOL (*p* = 0.047; Supplementary Table S4). There was a trend for an interaction of group and week (*p* = 0.083*;* Supplementary Table S4) whereby the MediDiet group at week 8 tended to report a lower impact on QOL for bodily discomfort than the control group (*p* = 0.084; Supplementary Table S4). There was a significant interaction for study week on the mobility dimension and PDQ-39 summary index scores (*p* = 0.039 and *p* = 0.038, respectively; Supplementary Table S4) as the control group reported decreased impact on QOL at week 8 when compared to their week 0 scores (*p* = 0.016; Supplementary Table S4). There was no effect of group or the interaction between the group and study week for other PDQ-39 dimensional scores (Supplementary Table S4).

Anxiety and depression scores overall were considered within the normal range at baseline (<17 and <7, respectively; Supplementary Table S5). There was a trend for an effect of study week on anxiety scores (*p* = 0.069; Supplementary Table S5) in which the reported level of anxiety decreased during the intervention period. The control group reported higher symptoms of depression than MediDiet (*p* = 0.031; Supplementary Table S5), but no effect of study week (*p* = 0.247) or group and week (*p* = 0.943) interactions were observed.

### 3.4. Intestinal Permeability and Inflammation

**Figure 6:**
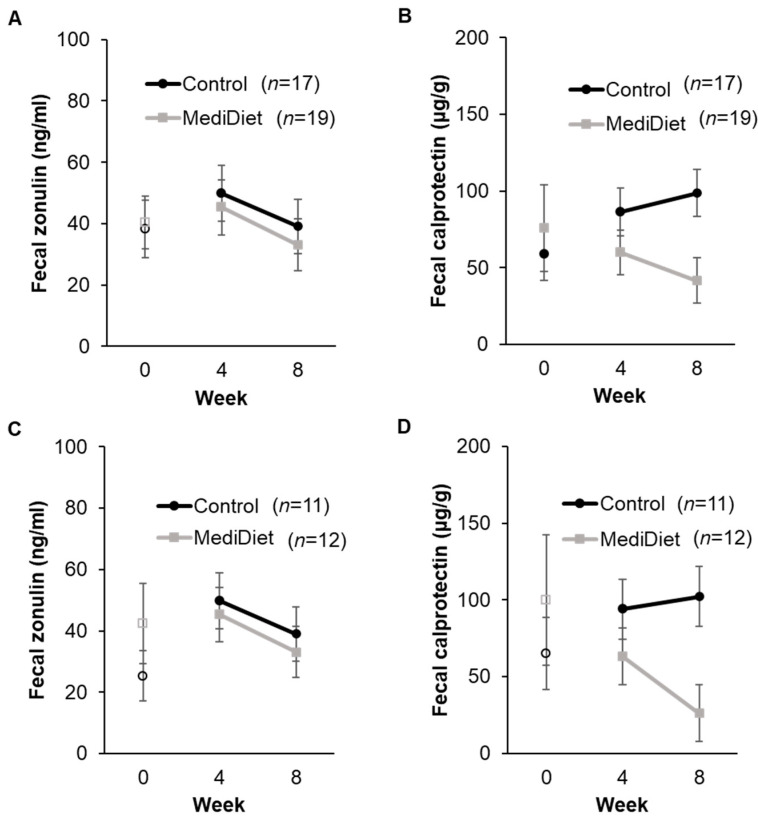
Fecal zonulin and calprotectin concentrations by study week in the intent-to-treat (**A**,**B**) and per protocol analyses (**C**,**D**). A generalized linear mixed model was used with log-transformed values, including only weeks 4 and 8 for analyses. Covariates were tested in the full model, and nonsignificant covariates were removed hierarchically, beginning with interactions with the largest *p*-values. Baseline values (week 0) were included as a covariate in final models and are shown as open circles and squares for the control and MediDiet groups, respectively. Baseline was compared using the Mann–Whitney U test and was not different between groups for fecal zonulin ((**A**), *p* = 0.609; (**C**), *p* = 0.56) or calprotectin ((**B**), *p* = 0.72; (**D**), *p* = 0.88). The interaction for study group was significant for fecal calprotectin during the study intervention ((**B**), *p* = 0.055; (**D**), *p* = 0.035) after adjusting for Hoehn and Yahr stage ((**B**), *p* = 0.011; (**D**), *p* = 0.012). *p-*values were adjusted using the post hoc Tukey–Kramer method for multiple comparisons. No differences were observed for all other outcomes. Values are untransformed least squares means ± SEM. Abbreviations: MediDiet, Mediterranean diet.

Fecal zonulin concentrations at baseline were low (<80 ng/mL) and not different between groups (ITT, *p* = 0.609; PP, *p* = 0.559; Figure 6A,C). Baseline values were included as a fixed effect in final models. No interactions for group (ITT, *p* = 0.334; PP, *p* = 0.520), study week (ITT, *p* = 0.141; PP, *p* = 0.572), or interaction of group and week (ITT, *p* = 0.294; PP, *p* = 0.490) were observed for fecal zonulin concentrations (Figure 6A,C).

Fecal calprotectin concentrations at baseline were elevated (>50 µg/g) and not different between groups (ITT, *p* = 0.727; PP, *p* = 0.878; Figure 6B,D). For ITT analyses, the MediDiet group tended to have lower concentrations than controls when adjusted for baseline (*p* = 0.055, Figure 6B). Fecal calprotectin differed with H&Y stage, whereby H&Y stage 1 was higher than stage 2 (*p* = 0.0110). For PP analyses, concentrations of fecal calprotectin were lower in the MediDiet group than the control group during the intervention period after adjusting for baseline and H&Y stage (*p* = 0.035; Figure 6D).

### 3.5. Microbial Communities

After removal of low-quality reads, a total of 3,586,154 sequence reads with an average of 39,846 reads/sample was obtained, providing sufficient sequencing depth for the analyses. During the study period, alpha diversity remained stable in both the control as well as the MediDiet groups (Supplementary Figure S2). No difference between the MediDiet and control groups was detected in overall beta diversity (Supplementary Figure S3), suggesting that the intervention had only minimal effects on overall microbiota composition.

## 4. Discussion

The aims of this study were to determine the effect of a MediDiet intervention on constipation symptoms and GI function, permeability, and inflammation in PwP who self-identified as having constipation symptoms. The primary aim examined the change from baseline in constipation symptoms in those instructed to eat a MediDiet and follow standard of care instructions for constipation versus those instructed on standard of care alone (control group). Constipation symptoms decreased with both the MediDiet and control interventions, but the change after the 8-week intervention was not different between groups. When symptoms were examined across weeks 0, 4, and 8 of the intervention, both groups reported lower constipation as well as abdominal pain syndrome scores, and there was a trend toward less use of laxatives. However, post hoc testing showed that only the MediDiet group had a significant decrease in GSRS constipation syndrome scores at weeks 4 and 8 as well as abdominal pain scores at week 8 when compared to their baseline. This may be attributed to the increase in MediDiet adherence and dietary fiber intake. At baseline, total dietary fiber content and density in both groups were lower than reported in other studies [68,69,70,71,72] but similar to the reported average intake in the United States [73]. Dietary fiber intake density was highest at week 4 in the MediDiet group and almost met the Dietary Guidelines for Americans recommendation to consume 14 g per 1000 kcal dietary fiber per day [74]. Despite the increased fiber intake in the MediDiet group, stool frequency and form remained unchanged across the study intervention. Because participants reported experiencing slight discomfort in constipation symptoms at baseline, it is possible a greater effect would be seen in those with more bothersome symptoms.

Few studies have explored dietary intake, let alone dietary interventions to improve constipation symptoms in PwP [30,75]. Our previously published 1-arm pilot study showed a decrease in constipation syndrome scores but no change in stool consistency or number with implementation of a MediDiet [36]. A systematic review and meta-analysis reported on four randomized controlled trials that examined probiotics versus various controls on the number of bowel movements per week in PwP [76]. Compared to the control group, probiotics increased the number of stools per week without improving stool consistency, but the quality of the evidence was low [76].

It was also postulated that the MediDiet intervention would decrease markers of intestinal permeability and inflammation by increasing intake of colorful fruits, vegetables, and legumes rich in fiber and phenolic compounds [21]. However, no differences in fecal zonulin were observed between groups. Results of this study are consistent with our pilot study, which showed no changes in intestinal permeability as measured by fecal zonulin and orally administered sugar probes [36]. Zonulin has been implicated as a possible biomarker of intestinal permeability, especially in conditions such as obesity and inflammatory bowel disease [77,78]. Further, a 3-month MediDiet intervention decreased fecal zonulin concentrations in women with increased intestinal permeability [27]. Lack of agreement exists on whether collecting serum or fecal samples is more accurate for measuring intestinal permeability and what is considered normal concentration ranges. Concentrations in the current study were lower than previously reported studies in PwP [16,79]. Recent studies have also disputed the specificity of commercially available ELISA kits, as these kits can detect additional members of the zonulin protein family [80,81]. Therefore, our findings may have been limited by our ability to detect changes in zonulin concentrations, and more specific assay methods with clinically relevant reference ranges should be developed in the future.

Fecal calprotectin, a reliable marker of intestinal inflammation, tended to be lower in the MediDiet group compared to controls for ITT participants. This effect was significant for PP participants, which suggests that participants with strong adherence to a MediDiet over 8 weeks had less intestinal inflammation. Fecal calprotectin is released by neutrophils, drives further neutrophil chemotaxis, and activates pattern recognition receptors (i.e., toll-like receptor 4) on innate and adaptive immune cells. This in turn promotes a proinflammatory environment in the GI tract [82]. To date, this is the first study to document a difference in fecal calprotectin concentrations using a dietary intervention in PwP.

Findings in this study are consistent with previous investigations in people with inflammatory bowel diseases, which demonstrated that strong adherence to a MediDiet is associated with lower concentrations of fecal calprotectin [83,84,85,86]. It is hypothesized that this mechanism is due to a higher intake of fiber-dense foods [87]. The MediDiet pattern promotes higher consumption of plant foods rich in fermentable dietary fibers and polyphenolic compounds. Fermentable dietary fibers may mediate intestinal inflammation by increasing the abundance of commensal microbes, such as *Faecalibacterium prausnitizii* and *Prevotella*, and the production of SCFAs, both of which are reduced in Parkinson’s [28,32,35,88]. SCFAs produced by commensal microbes promote proliferation of anti-inflammatory regulatory T cells and immune tolerance [89]. Polyphenolic compounds may attenuate intestinal inflammation by serving as an antioxidant or a substrate for commensal microbe fermentation [19,90,91]. Unfortunately, taxonomic changes at the genera and species level that would support this mechanism were not explored in the current study and should be explored in the future. It was also observed that fecal calprotectin concentrations inversely correlated with H&Y stage. This was an unexpected finding, as it has been hypothesized that disease progression is associated with increased intestinal inflammation [92]. It is possible this association was only statistically significant and not clinically relevant, as only a small number of participants (*n* = 5) were H&Y stage 1 and no PwP with H&Y stages ≥3 were included in the present study. Nevertheless, this is the first study to report the effect of a dietary intervention on markers of inflammation in early Parkinson’s disease and the relationship with H&Y stage.

Microbial diversity remained stable throughout the intervention period for both groups. This finding is consistent with previous literature, which has demonstrated short-term changes in dietary intake have minimal effects on inter-individual variability of host microbial profiles [93,94]. It is hypothesized that a longer intervention may be needed to show effects on microbial diversity and functional metabolism of the microbial metabolome. However, results have been mixed using up to a 1-year MediDiet intervention [95,96]. The mechanism by which a MediDiet is associated with microbial changes in Parkinson’s should be explored further using functional profiling (i.e., shotgun metagenomic sequencing), as others have postulated that the pathophysiology of the disease may be impacted through crosstalk along the gut–brain axis. Changes in relative and absolute abundances of taxa were not explored in the current study, which may have offered insight into whether smaller, relevant taxonomic changes occur with a short-term MediDiet intervention in PwP.

It was anticipated that there would be a decrease in body weight and BMI in the MediDiet group due to an increase in whole foods and a reduction in total energy intake [36]. Body weight remained similar in both groups across the study intervention, and while BMI was statistically different for study group and week, this may not be clinically significant given the change from baseline values (<0.5 points) was minimal. No differences were observed in body composition or handgrip strength outcomes. Based on the results of this study, it appears that PwP are able to maintain their nutritional status with a MediDiet. Additionally, QOL scores were lower in this study population than reported previously and consistent with low reported symptoms of anxiety and depression [97,98]. This is likely due to excluding advanced PD, as a higher H&Y stage is associated with higher PDQ-39 summary index scores [99]. While there was an improvement in the mobility dimension and summary index score for the control group, it is possible this was a statistically significant but not clinically significant finding given low reported scores. The MediDiet group reported a lower impact on QOL for bodily discomfort than controls, as well as an improvement at week 8 when compared to their baseline, which is in agreement with this group also reporting decreased symptoms of abdominal pain at week 8. It is hypothesized that the increase in dietary fiber and improvement in constipation symptoms led to a decrease in abdominal pain experienced in the MediDiet group.

Participant compliance with diet recommendations may have influenced our ability to detect differences in outcomes for ITT analyses. At weeks 4 and/or 8, adherence to a MediDiet was low in seven participants in the MediDiet group and high in three participants in the control group. Despite this, adherence to diet recommendations remained strong, likely due to weekly telephone calls and self-monitoring of food intake with questionnaires [100]. It is possible a longer intervention period would have influenced the results of this study and could be used in the future to evaluate the progression of Parkinson’s motor and non-motor symptoms.

An unforeseen limitation was the impact of the COVID-19 pandemic on study recruitment. Participants who were unable to travel (*n* = 11) to the study site conducted all visits virtually and self-reported their weight. This may have influenced adherence to diet recommendations and self-reporting bias, particularly for body weight measurements. Despite this, virtual interventions have been associated with behavior change in previous studies, and other outcome measures such as online questionnaires and stool sample collection were performed similarly between those who attend in-person vs. virtual visits [101,102]. Participants were reminded regularly to weigh after fasting and wear similar clothes before each virtual visit to reduce self-reporting bias and variation in the present study. Additionally, because some secondary measures were unable to be completed in person (i.e., MDS-UPDRS, MoCA, body composition, and handgrip strength), this may have reduced our power to detect differences between groups. Future studies that include virtual study visits should consider using questionnaires that are validated to be performed virtually and/or providing anthropometric devices that can remotely report data to limit self-reporting bias. Another unexpected limitation was lower MoCA scores in the MediDiet group at baseline. Questionnaire compliance was high in both groups (>80% completed); however, some participants (MediDiet, *n* = 3; control, *n* = 2) anecdotally reported needing some assistance from another person to complete questionnaires. This may have confounded the validity of some questionnaire data and our ability to detect differences between groups. Future research should consider baseline cognitive status as a criterion for participation in studies, particularly when methods rely on participant recall. Another limitation of this study was the small sample size for our PP primary outcome, which likely reduced the power to observe differences in constipation symptoms.

A strength of this study was the use of standard of care for constipation symptoms as a comparator group. This design allowed us to determine whether the MediDiet plus standard of care was more effective than standard of care alone. Although both interventions similarly decreased constipation symptoms, the MediDiet intervention provided additional dietary and GI benefits.

## 5. Conclusions

The MediDiet and standard of care interventions were effective strategies for reducing constipation symptoms in PwP who typically report symptoms related to constipation; however, only the MediDiet provided additional benefit of increased dietary fiber intake and less intestinal inflammation. The mechanism by which a MediDiet is associated with lower intestinal inflammation should be further explored, as well as how this diet influences microbial composition, systemic inflammation, and crosstalk along the gut–brain axis. Overall, there is a need for long-term, randomized interventional studies using a MediDiet for PwP to understand the effects of this diet on Parkinson’s disease progression and treatment of motor and non-motor symptoms.

## Figures and Tables

**Table 1:** Baseline demographic and disease-related variables of participants by study group.

Characteristics	Control (*n* = 17)	MediDiet (*n* = 19)	*p*-Value ^1^
Age (y)	65.1 ± 2.2	68.8 ± 1.4	0.29
Sex, *n* (%)			
Male	10 (59)	12 (63)	1.00
Female	7 (41)	7 (37)	
Race, *n* (%)			
White	16 (94)	19 (100)	0.47
Asian	1 (6)	-	
Ethnicity, *n* (%)			
Non-Hispanic or Latino	16 (94)	17 (89)	1.00
Hispanic or Latino	1 (6)	2 (11)	
MDS-UPDRS Scores ^2^			
Part I	11.6 ± 1.5	8.9 ± 1.6	0.21
Part II	12.4 ± 9.0	8.1 ± 5.7	0.27
Part III	22.4 ± 3.1	24.6 ± 1.7	0.38
Part IV	3.9 ± 0.7	2.1 ± 0.8	0.11
Total Score	50.3 ± 6.5	43.7 ± 3.9	0.62
Hoehn and Yahr Stage			
0–1.5	2 (12)	3 (16)	1.00
2–2.5	15 (88)	16 (84)	
MoCA Score ^2^	27.4 ± 0.5	25.2 ± 0.8	0.02
Disease duration (y)	4.8 ± 0.6	5.0 ± 1.1	0.45
LEDD (mg)	729 ± 107	661 ± 97	0.73

Values represent means ± SEM. ^1^ Continuous variables were checked for normality and heterogeneity and then analyzed using the Mann–Whitney U test. Categorical variables were analyzed using Fisher’s exact test to determine differences between groups. ^2^ Participants who attended in-person study visits (Control, *n* = 14 and MediDiet, *n* = 11) were evaluated while fasting during the clinical ‘ON’ state. Abbreviations: LEDD, levodopa equivalent daily dose; MDS-UPDRS, Movement Disorder Society Unified Parkinson’s Disease Rating Scale; MediDiet, Mediterranean diet; MoCA, Montreal Cognitive Assessment.

**Table 2:** Laxative usage, stool frequency and form, and digestion-associated quality of life by study group and week.

	Week 0		Week 4		Week 8		*p*-Value ^1^		
	Control	MediDiet	Control	MediDiet	Control	MediDiet	Group	Week	Group x Week
Intent-to-treat ^2^									
Laxative usage (d/wk) ^3^	1.76 ± 0.56	1.42 ± 0.53	1.29 ± 0.56	0.68 ± 0.53	1.06 ± 0.56	0.58 ± 0.53	0.044	0.055	0.473
Stool frequency (BM/wk) ^4^	6.41 ± 1.21	7.53 ± 1.15	8.24 ± 1.21	6.37 ± 1.15	8.35 ± 1.21	7.16 ± 1.15	0.396	0.361	0.870
Hard stools (%) ^5^	27.8 ± 7.5	40.3 ± 9.1	30.2 ±8.2	27.9 ± 8.9	34.6 ± 7.2	33.2 ± 9.7	0.185	0.746	0.756
DQLQ score ^6^	1.23 ± 0.29	0.83 ± 0.27	1.32 ± 0.29	0.88 ± 0.28	1.29 ± 0.29	0.51 ± 0.27	0.806	0.233	0.435
Per protocol ^7^									
Laxative usage (d/wk) ^8^	2.18 ± 0.82	2.08 ± 0.78	1.55 ± 0.82	1.08 ± 0.78	1.27 ± 0.82	0.83 ± 0.78	0.043	0.069	0.318
Stool frequency (BM/wk)	6.36 ± 1.34	6.75 ± 1.28	8.82 ± 1.34	6.00 ± 1.28	7.82 ± 1.34	6.25 ± 1.28	0.268	0.314	0.547
Hard stools (%)	36.3 ± 10.5	42.1 ± 10.3	29.9 ± 11.4	24.6 ± 10.2	35.5 ± 10.5	36.0 ± 11.6	0.683	0.921	0.693
DQLQ score	0.93 ± 0.36	0.71 ± 0.34	0.80 ± 0.36	0.99 ±0.34	0.71 ± 0.35	0.60 ± 0.34	0.280	0.246	0.408

Values are means ± SEM. ^1^ A generalized linear mixed model was used with log-transformed values for analyses and adjusted using the post hoc Tukey–Kramer method for multiple comparisons. Covariates were tested in the full model, and nonsignificant covariates were removed hierarchically, beginning with interactions with the largest *p*-values. *p-*values were adjusted using the post hoc Tukey–Kramer method for multiple comparisons. ^2^ The number of participants included in the ITT analysis was *n* = 19 for MediDiet and *n* = 17 for control groups. ^3^ Age is included in the model as a covariate (*p* = 0.020). ^4^ MET min were included in the ITT and PP models (*p* = 0.011 and *p* = 0.021, respectively). ^5^ The percentage of hard stools reflects the percentage of stools that were scored as type 1 or 2 using the BSFS, where scores range from type 1 (hard lumps) to type 7 (watery). MET min were included in the ITT and PP models (*p* = 0.037 and *p* = 0.318, respectively). ^6^ DQLQ scores range from 0 = better to 9 = worse digestion-associated quality of life. Perceived stress was included in the ITT and PP models (*p* = 0.044 and *p* = 0.282, respectively). ^7^ The number of participants included in PP analysis was *n* = 12 for MediDiet and *n* = 11 for control groups. ^8^ Sex and Hoehn and Yahr stages were included in the model (*p* = 0.045 and *p* = 0.017, respectively). Abbreviations: BM, bowel movement; BSFS, Bristol Stool Form Scale; DQLQ, digestion-associated quality of life; ITT, intent-to-treat; MediDiet, Mediterranean diet; MET, metabolic equivalent task; PP, per protocol.

## Data Availability

The raw data supporting the conclusions of this article will be made available by the authors and the University of Florida on request.

## Supplementary Materials

The following supporting information can be downloaded at: https://www.mdpi.com/article/10.3390/nu16172946/s1. Methods for Exploratory Outcomes. Figure S1: Body weight and BMI by study week. Figure S2: Alpha diversity distribution by group and time. Figure S3: PCoA based on Bray Curtis Distance by interventions with all timepoints included. Table S1: Mean syndrome scores from the Gastrointestinal Symptom Rating Scale by study week. Table S2: Mean dietary intake between interventions by study week. Table S3: Body composition and handgrip strength by study week. Table S4: PDQ-39 dimensional and overall quality of life scores by intervention and study week. Table S5: Anxiety and depression scores by intervention and study week.
