# Supplementary material for: Promotion of a Mediterranean Diet Alters Constipation Symptoms and Fecal Calprotectin in People with Parkinson’s Disease: A Randomized Controlled Trial
Source: Nutrients. 2024 Sep 2;16(17):2946. doi: 10.3390/nu16172946 (PMC11396875; doi:10.3390/nu16172946)
Supplement: Supplementary file 1 [file nutrients-16-02946-s001.zip › nutrients-3161706-supplementary.pdf]

Rusch, C, et al. Promotion of a Mediterranean diet alters constipation symptoms and fecal calprotectin in people with Parkinson's disease: a randomized controlled trial.

SUPPLEMENTARY MATERIAL

METHODS for EXPLORATORY OUTCOMES

Protocol details of the study have been previously described in detail (1).

Statistical Methods

Nutritional Status and Body Composition Analyses

A generalized linear mixed model (GLMM) was used to analyze body weight, BMI, body composition and handgrip strength) on an intent-to-treat (ITT) basis. Additional covariates were included in the full models with nonsignificant covariates removed hierarchically beginning with interactions with the largest *P*-values. Age was trending significant (*P*=0.0508) and retained with sex (*P*=0.0005) as covariates for handgrip strength in the final model. Body weight and BMI were analyzed on both an ITT and per protocol (PP) basis in all eligible participants. Body weight was self-reported in 11 virtual group participants (MediDiet group, n=8; control group, n=3) and missing at week 4 in 1 control group participant. Body composition and handgrip measurements were conducted in participants who attended in-person visits (MediDiet group, n=11; control group, n=14). Two participants in the control group were excluded from body composition measurements due to due to implanted devices. Handgrip strength and body composition were missing at week 4 in 2 participants from each group.

Exploratory Questionnaires

Exploratory questionnaires including the Parkinson's Disease Questionnaire-39 (PDQ-39), Hamilton Anxiety Scale (HAM-A) and Hamilton Depression Scale (HAM-D) were analyzed at weeks 0, 4 and 8 using GLMM. PDQ-39 scores were analyzed by dimension. Dimension scores were based on the sum of scores from 3 to 10 questions divided by the maximum possible score multiplied by 100. For overall QOL, a summary index score (sum of dimension total scores divided by eight) was calculated. PDQ-39 scores were square root transformed to meet assumptions for normality. HAM-A and HAM-D are shown as total scores. No covariates were included in analyses given these were exploratory outcomes.

1. Rusch C, Beke M, Tucciarone L, Dixon K, Nieves C, Jr., Mai V, et al. Effect of a Mediterranean diet intervention on gastrointestinal function in Parkinson's disease (the MEDI-PD study): study protocol for a randomised controlled trial. BMJ Open. 2021;11(9):e053336. doi: 10.1136/bmjopen-2021-053336.

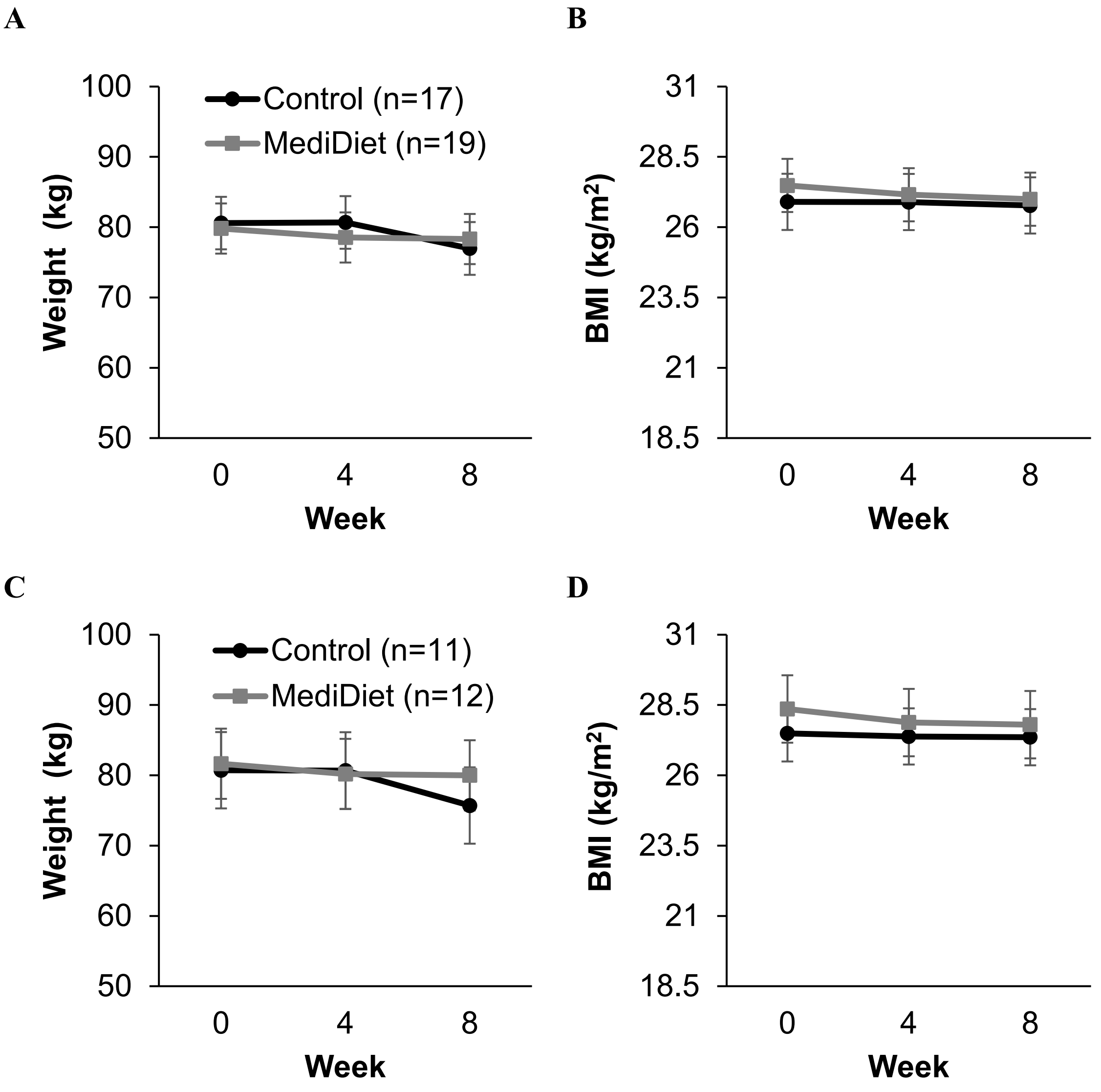

Figure S1. Body weight and BMI by study week in the intent-to-treat analyses (**A, B**) and per protocol analyses (**C, D**). A generalized linear mixed model was used for analyses with group, week and interaction of group and week in the models. Covariates were tested in the full model and nonsignificant covariates were removed hierarchically beginning with interactions with the largest *P*-values. *P*-values were adjusted using post-hoc Tukey-Kramer method for multiple comparisons. Body weight differed with sex (**A**, *P*<0.001; **C**; *P*=0.006) and was included as a covariate The final model for body weight included group (**A**, *P*=0.916; **C**; *P*=0.822), week (**A**, *P*=0.139; **C**; *P*=0.248), and interaction of group and week (**A**, *P*=0.213; **C**; *P*=0.329). BMI was significant for study week (**B**, *P*<0.001; **D**, *P*=0.001) and interaction of group and week (**B**, *P*=0.016; **D**, *P*=0.044) but not group (**B**, *P*=0.798; **D**, *P*=0.732) in the final model. Values are least squares means ± SEM. BMI; body mass index; MediDiet, Mediterranean diet.

Rusch, C, et al. Promotion of a Mediterranean diet alters constipation symptoms and fecal calprotectin in people with Parkinson's disease: a randomized controlled trial.

A

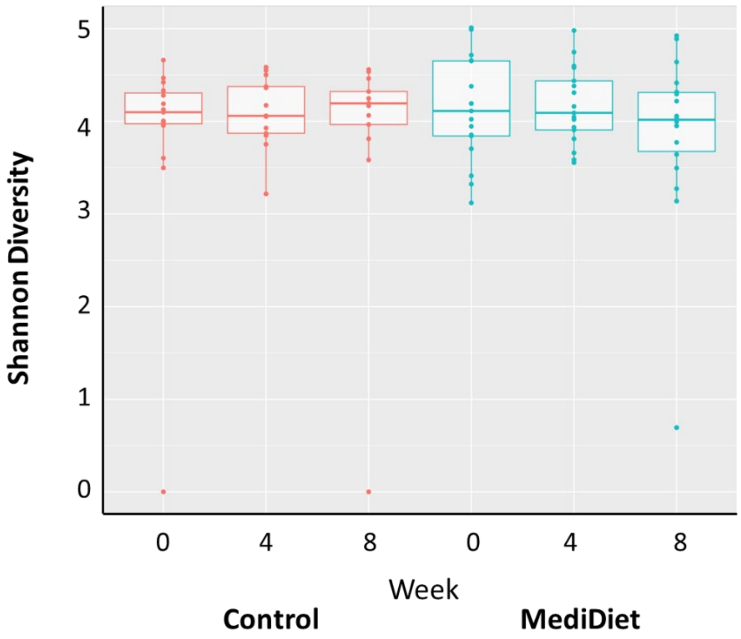

B

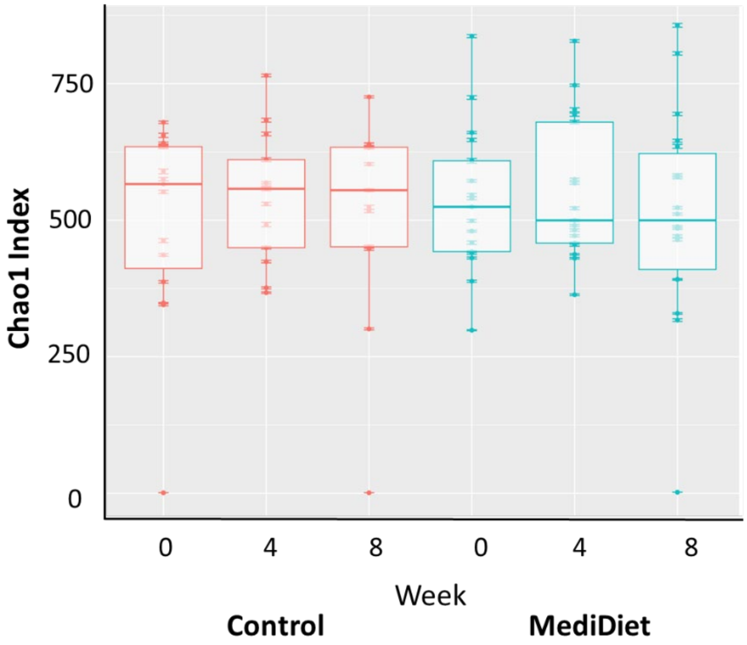

Figure S2. Alpha diversity distribution as measured by **A**) Shannon and **B**) Chao1 for control (n=17) and MediDiet (n=19) interventions and time points. No significant differences were found between groups or time points.

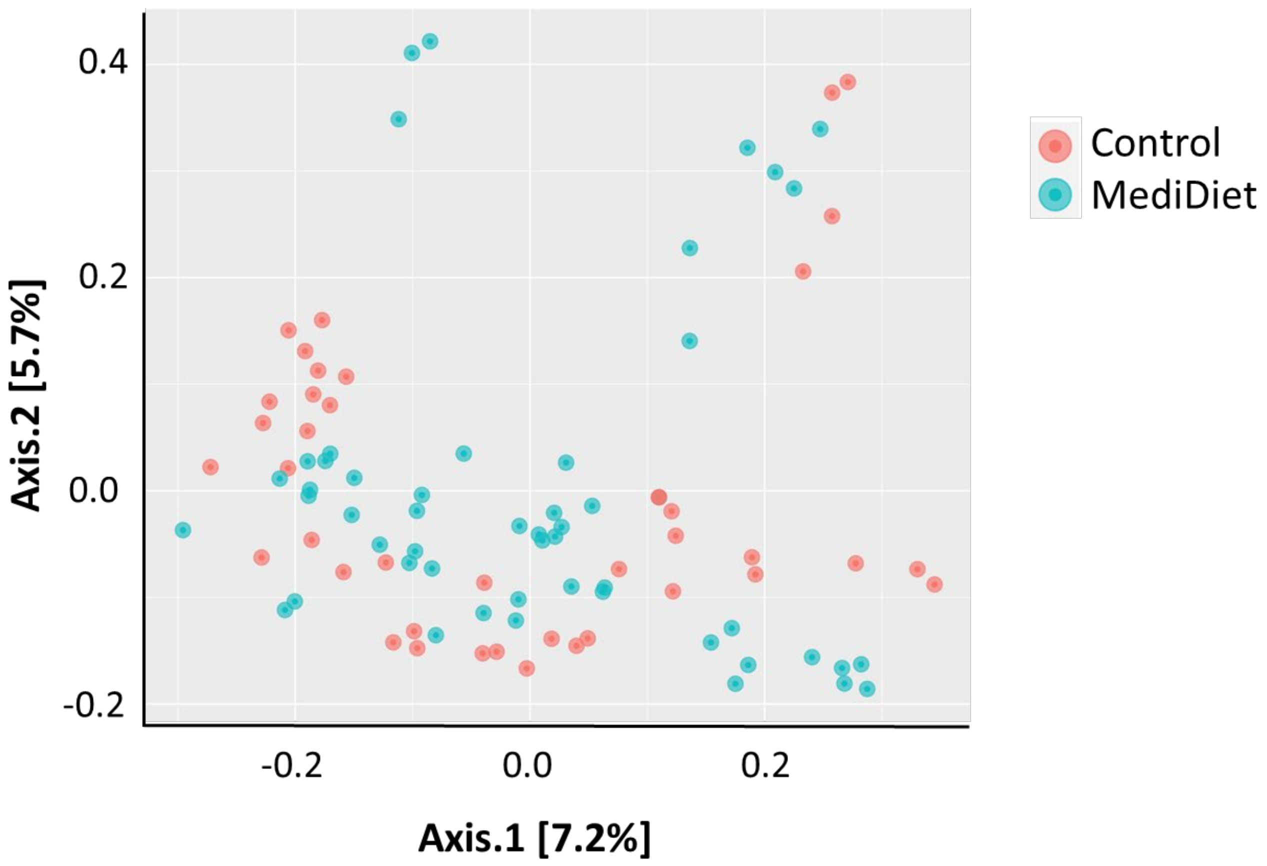

Figure S3. PCoA based on Bray Curtis Distance by control (n=17) and MediDiet (n=19) interventions with all timepoints included. No significant difference was found between interventions.

Table S1. Mean syndrome scores from the Gastrointestinal Symptom Rating Scale by study week.

	Week 0		Week 4		Week 8		*P*-value[1]		
	Control	MediDiet	Control	MediDiet	Control	MediDiet	Group	Week	Group x Week
**Intent-to-treat[2]**									
Reflux[3]	1.53 ± 0.20	1.18 ± 0.09	1.25 ± 0.10	1.06 ± 0.04	1.27 ± 0.08	1.08 ± 0.06	0.054	0.049	0.441
Indigestion[4]	2.25 ± 0.28	1.71 ± 0.12	2.20 ± 0.24	1.58 ± 0.14	2.02 ± 0.23	1.65 ± 0.13	0.100	0.418	0.654
Diarrhea[5]	1.50 ± 0.21	1.42 ± 0.11	1.50 ± 0.25	1.19 ± 0.09	1.47 ± 0.23	1.16 ± 0.09	0.388	0.159	0.501
**Per protocol[6]**									
Reflux	1.20 ± 0.08	1.21 ± 0.13	1.10 ± 0.07	1.09 ± 0.06	1.09 ± 0.06	1.13 ± 0.09	0.790	0.471	0.787
Indigestion	2.10 ± 0.28	1.67 ± 0.13	1.88 ± 0.24	1.50 ± 0.20	1.64 ± 0.18	1.65 ± 0.17	0.404	0.055	0.198
Diarrhea	1.30 ± 0.27	1.53 ± 0.16	1.30 ± 0.24	1.18 ± 0.12	1.21 ± 0.21	1.06 ± 0.04	0.772	0.040	0.176

Values are means ± SEM.
[1]A generalized linear mixed model was used with log-transformed values for analyses and adjusted using post-hoc Tukey-Kramer method for multiple comparisons. Covariates were tested in the full model and nonsignificant covariates were removed hierarchically beginning with interactions with the largest *P*-values. Symptom scores range from 1=no discomfort to 7=very severe discomfort.
[2]Number of participants included in ITT analyses was n=19 for MediDiet and n=17 for control groups.
[3]Reflux symptoms include heartburn and acid regurgitation. Sex was included in the ITT model and trended toward being higher for females (*P*=0.084).
[4]Indigestion symptoms include rumbling, bloating, burping, and gas.
[5]Diarrhea symptoms include diarrhea, loose stools, and urgent need for defecation.
[6]Number of participants included in PP analysis was n=12 for MediDiet and n=11 for control groups.
Abbreviations: ITT, intent-to-treat; MediDiet, Mediterranean diet; PP, per protocol.

Table S2. Mean dietary intake between interventions by study week.

	Week 0		Week 4		Week 8		*P*-values[1]		
Dietary Components[2]	Control (n=17)	MediDiet (n=19)	Control (n=17)	MediDiet (n=19)	Control (n=17)	MediDiet (n=19)	Group	Week	Group x Week
Total kcal	2044 ± 143	2019 ± 154	2061 ± 129	1893 ± 148	2189 ± 168	2010 ± 151	0.369	0.413	0.754
Protein (g)	78.8 ± 8.5	80.7 ± 6.8	85.9 ± 6.3	83.5 ± 5.5	79.1 ± 5.5	88.2 ± 5.0	0.056	0.140	0.140
Carbohydrates (g)	225 ± 15	211 ± 16	226 ± 15	195 ± 20	248 ± 17	208 ± 19	0.081	0.702	0.675
Dietary fiber (g)	17.8 ± 1.6	17.5 ± 1.5	20.0 ± 1.5	25.0 ± 2.5†	20.2 ± 2.0	23.2 ± 2.0	0.018	<0.001	0.004
Total sugars (g)	102 ± 9	93.5 ± 11	97 ± 6	87 ± 11	114 ± 10	91 ± 10	0.234	0.581	0.319
Total fat (g)	88.6 ± 6.7	90.0 ± 8.3	86.1 ± 4.9	87.1 ± 7.5	95.8 ± 8.6	90.1 ± 8.6	0.185	0.963	0.277
Saturated fat (g)	27.4 ± 2.2	30.9 ± 3.2	26.8 ± 1.6	21.5 ± 1.8‡	30.6 ± 3.0	24.4 ± 2.7‡	0.618	0.002	0.004
MUFA (g)	30.1 ± 2.2	31.8 ± 3.0	30.2 ± 1.9	37.7 ± 4.0†	33.2 ± 3.1	36.1 ± 4.0	<0.001	0.011	0.007
Oleic acid (g)	28.1 ± 2.1	29.5 ± 2.8	28.1 ± 1.7	35.7 ± 3.8†	31.1 ± 2.9	34.0 ± 3.9	0.001	0.008	0.004
PUFA (g)	23.5 ± 2.4	19.5 ± 1.8	21.3 ± 1.6	21.4 ± 2.1	23.6 ± 2.3	22.6 ± 2.1	0.842	0.690	0.019
Linoleic acid (g)	20.6 ± 2.1	17.0 ± 1.6	18.5 ± 1.4	18.8 ± 1.8	20.5 ± 2.0	19.8 ± 1.9	0.918	0.783	0.012
ALA (g)	2.4 ± 0.3	1.9 ± 0.2	2.2 ± 0.2	2.0 ± 0.2	2.6 ± 0.3	2.1 ± 0.2	0.229	0.400	0.224
EPA (mg)	0.06 ± 0.01	0.08 ± 0.03	0.06 ± 0.03	0.08 ± 0.02	0.05 ± 0.02	0.10 ± 0.02	0.143	0.916	0.621
DHA (mg)	0.10 ± 0.02	0.15 ± 0.05	0.12 ± 0.05	0.16 ± 0.03	0.11 ± 0.03	0.23 ± 0.04	0.076	0.392	0.347
Water (ml)	2418 ± 328	2033 ± 211	2404 ± 304	2569 ± 325	2420 ± 315	2390 ± 227	0.823	0.208	0.180

Values represent means ± SEM.

[1]Analyzed on an intent-to-treat basis. *P*-values calculated using least square means with group, week, kcal, and interaction of group and week in the final generalized linear mixed model (with exception of total kcal which included sex [*P*=0.0002] and water which included no covariates) and adjusted using post-hoc Tukey-Kramer method for multiple comparisons. †*P*-values <0.01 compared between control vs. MediDiet groups at specified week. ‡*P*-values <0.001 compared to week 0 in the MediDiet group.

[2]Mean dietary intake represents average of four 24-hour dietary recalls (2 weekday, 2 weekend) for each time point using the Automated Self-Administered 24-hour Dietary Assessment Tool, version 2018.

Abbreviations: ALA, alpha-linolenic acid; EPA, eicosapentaenoic acid; DHA, docosahexaenoic acid; MediDiet, Mediterranean diet; MUFA, monounsaturated fatty acids; PUFA, polyunsaturated fatty acids.

Table S3. Body composition and handgrip strength by study week.

	Week 0		Week 4		Week 8		*P*-values[1]		
Measurement	Control (n=12)	MediDiet (n=11)	Control (n=11)	MediDiet (n=10)	Control (n=11)	MediDiet (n=12)	Group	Week	Group x Week
Body Composition[2]									
FFM (%)	69.0 ± 2.5	66.2 ± 3.2	72.3 ± 2.2	66.6 ± 3.8	70.0 ± 2.4	65.6 ± 3.4	0.261	0.348	0.678
FM (%)	31.0 ± 2.5	33.8 ± 3.2	27.7 ± 2.2	33.4 ± 3.8	30.0 ± 2.4	34.2 ± 3.4	0.261	0.348	0.678
TBW (L)	42.6 ± 3.2	38.8 ± 2.8	44.6 ± 3.5	39.4 ± 2.9	42.4 ± 2.5	37.5 ± 2.8	0.228	0.253	0.361
Handgrip strength[3] (kg)	34.2 ± 3.1	28.7 ± 3.3	35.4 ± 3.3	29.7 ± 3.8	36.2 ± 2.8	29.1 ± 3.0	0.718	0.834	0.962

Values represent means ± SEM.
[1]Analyzed on an intent-to-treat basis. *P*-values calculated using least square means with group, week and interaction of group and week in the final generalized linear mixed model (with exception of handgrip strength which included age [*P*=0.051] and sex [*P*<0.001] as covariates) and adjusted using post-hoc Tukey-Kramer method for multiple comparisons.
[2]FFM, FM and TBW were calculated using bioelectrical impedance spectroscopy after a 12-hour fast.
[3]Handgrip strength was measured using a hand dynamometer by taking the highest value of the dominant hand after 3 measurements at least 10 seconds apart. Handgrip strength included 11 participants in the MediDiet group and 14 participants in the control group.
Abbreviations: FFM, fat-free mass; FM, fat mass; MediDiet, Mediterranean diet; TBW; total body water.

Table S4. PDQ-39 dimensional and overall quality of life scores by interventions and study week.

	Week 0		Week 4		Week 8		*P*-values[1]		
Dimensional scores[2]	Control (n=15)	MediDiet (n=18)	Control (n=16)	MediDiet (n=19)	Control (n=17)	MediDiet (n=19)	Group	Week	Group x Week
Mobility	17.3 ± 4.8	12.2 ± 3.4	13.4 ± 3.0	12.0 ± 3.2	10.1 ± 3.1[‡]	12.2 ± 3.8	0.832	0.039	0.064
Activities of daily living	18.3 ± 5.2	13.2 ± 2.4	16.9 ± 4.3	13.2 ± 2.1	15.0 ± 4.6	14.5 ± 2.9	0.727	0.469	0.361
Emotional well-being	17.5 ± 4.6	15.3 ± 2.9	17.7 ± 4.2	17.3 ± 3.7	14.0 ± 3.9	16.2 ± 3.2	0.737	0.325	0.859
Stigma	15.0 ± 4.2	8.3 ± 3.0	11.7 ± 3.2	5.9 ± 1.9	11.8 ± 3.2	8.55 ± 2.8	0.098	0.253	0.505
Social support	15.6 ± 6.0	8.3 ± 3.4	13.0 ± 4.4	9.7 ± 4.0	10.8 ± 3.6	8.3 ± 3.2	0.396	0.831	0.518
Cognition	25.0 ± 3.7	18.4 ± 3.5	22.3 ± 2.7	21.7 ± 3.3	16.5 ± 2.7	20.4 ± 3.2	0.837	0.120	0.124
Communication	22.8 ± 5.9	15.3 ± 3.5	22.4 ± 5.7	16.2 ± 3.4	20.1 ± 4.8	14.5 ± 3.5	0.483	0.520	0.436
Bodily discomfort	37.8 ± 5.4	24.1 ± 4.0	32.8 ± 4.9	25.0 ± 4.1	33.8 ± 4.0	18.4 ± 3.5[§]	0.047	0.226	0.083
PDQ-39 SI	21.1 ± 3.2	14.4 ± 2.1	18.7 ± 2.4	15.1 ± 2.0	16.5 ± 2.4[‡]	14.1 ± 1.8	0.245	0.038	0.062

Values represent means ± SEM.

[1]Analyzed on an intent-to-treat basis. *P*-values were calculated using square root transformed LS-means with group, week, and interaction of group and week in the final generalized linear mixed model and adjusted using post hoc Tukey-Kramer method for multiple comparisons. ‡*P*-value <0.05 compared to week 0 in the control group. [§]*P*=0.084 between MediDiet and control group at week eight.

[2]Dimensional scores represent how often an individual experienced difficulties due to Parkinson's disease over the past month based on responses to 39 questions evaluating the eight dimensions with a higher score representing poorer quality of life. PDQ-39 SI scores represent overall quality of life as the sum of dimension total scores divided by eight.

Abbreviations: MediDiet, Mediterranean diet; PDQ-39, Parkinson's disease quality of life questionnaire; SI, Summary Index.

Table S5. Anxiety and depression scores by intervention and study week.

Questionnaire	Week 0 Control (n=17)	Week 0 MediDiet (n=19)	Week 4 Control (n=17)	Week 4 MediDiet (n=19)	Week 8 Control (n=17)	Week 8 MediDiet (n=19)	*P*-values[1] Group	*P*-values[1] Week	*P*-values[1] Group x Week
Anxiety[2]	7.35 ± 1.19	5.79 ± 0.79	6.81 ± 1.05	5.63 ± 0.86	5.88 ± 1.10	4.42 ± 0.81	0.239	0.069	0.745
Depression[3]	5.12 ± 0.68	3.37 ± 0.75	5.06 ± 0.82	3.05 ± 0.66	3.94 ± 0.87	2.63 ± 0.58	0.031	0.247	0.943

Values represent means ± SEM.

[1]*P*-values were calculated on an intent-to-treat basis using square root transformed LS-means with group, week, and group*week interactions in the final generalized linear mixed model and adjusted using post hoc Tukey-Kramer method for multiple comparisons.

[2]Anxiety scores were determined by the Hamilton Anxiety scale where scores between 0 to 17 are accepted within normal range.

[3]Depression scores were determined by the Hamilton Depression scale where scores between 0 to 7 are accepted as within normal range.

Abbreviation: MediDiet, Mediterranean diet
